# Culturable prokaryotic diversity of deep, gas hydrate sediments: first use of a continuous high-pressure, anaerobic, enrichment and isolation system for subseafloor sediments (DeepIsoBUG)

**DOI:** 10.1111/j.1462-2920.2009.02018.x

**Published:** 2009-12

**Authors:** R John Parkes, Gerard Sellek, Gordon Webster, Derek Martin, Erik Anders, Andrew J Weightman, Henrik Sass

**Affiliations:** 1 School of Earth and Ocean Sciences, Cardiff UniversityMain Building, Park Place, Cardiff, CF10 3AT, Wales, UK; 2 Cardiff School of Biosciences, Cardiff UniversityMain Building, Park Place, Cardiff, CF10 3AT, Wales, UK; 3 Institut für Mechanik, LKM, Technische Universität Berlin10587 Berlin, DE

## Abstract

Deep subseafloor sediments may contain depressurization-sensitive, anaerobic, piezophilic prokaryotes. To test this we developed the DeepIsoBUG system, which when coupled with the HYACINTH pressure-retaining drilling and core storage system and the PRESS core cutting and processing system, enables deep sediments to be handled without depressurization (up to 25 MPa) and anaerobic prokaryotic enrichments and isolation to be conducted up to 100 MPa. Here, we describe the system and its first use with subsurface gas hydrate sediments from the Indian Continental Shelf, Cascadia Margin and Gulf of Mexico. Generally, highest cell concentrations in enrichments occurred close to *in situ* pressures (14 MPa) in a variety of media, although growth continued up to at least 80 MPa. Predominant sequences in enrichments were *Carnobacterium*, *Clostridium*, *Marinilactibacillus* and *Pseudomonas*, plus *Acetobacterium* and *Bacteroidetes* in Indian samples, largely independent of media and pressures. Related 16S rRNA gene sequences for all of these *Bacteria* have been detected in deep, subsurface environments, although isolated strains were piezotolerant, being able to grow at atmospheric pressure. Only the *Clostridium* and *Acetobacterium* were obligate anaerobes. No *Archaea* were enriched. It may be that these sediment samples were not deep enough (total depth 1126–1527 m) to obtain obligate piezophiles.

## Introduction

Prokaryotes play a major role in global biogeochemical cycles (e.g. Jorgensen, 2000) due to their remineralization of dead biomass. Recently, however, the significance of their own biomass to the global total has also been recognized and surprisingly, considering their position towards the end of the photosynthetic food chain, they represent between 60% and 100% of the estimated total carbon in plants (Whitman *et al*., 1998). The vast majority of this prokaryotic biomass is in subsurface environments, *c.* 90% (Whitman *et al*., 1998), with the dominant habitat being marine sediments (Parkes *et al*., 1994). Only a tiny percentage of microscopically detected prokaryotic cells can be cultured from subseafloor sediments (e.g. 0.1%, D'Hondt *et al*., 2004) even though a high proportion of microscopic cells are viable (up to at least ∼30%, Schippers *et al*., 2005) and there are significant concentrations of intact polar membrane lipids, which are biomarkers for live cells (Zink *et al*., 2003). In addition, there is a large discrepancy between the prokaryotic types present in 16S rRNA gene clone libraries and isolated organisms. For example, in Peru Margin subseafloor sediments *Gammaproteobacteria* and green non-sulfur bacteria (*Chloroflexi*) dominated the bacterial clone libraries (Parkes *et al*., 2005), while *Firmicutes* and *Alphaproteobacteria* were the dominant cultured groups (D'Hondt *et al*., 2004, Batzke *et al*., 2007). Clone libraries also contain many phylotypes unrelated to cultured sequences (Fry *et al*., 2008). Therefore, there is a large prokaryotic diversity in subseafloor sediments with no cultured representatives, which severely limits our understanding of this major global habitat.

A key aspect of subsurface environments is elevated pressure, e.g. ∼70% of the ocean is at a pressure of 38 MPa or above (Abe and Horikoshi, 2001), plus there is up to 10 km (∼100 MPa) of sediment in some locations. Thus, the majority of prokaryotes on earth live under and are likely to be adapted to high pressure, which could be essential for isolation of representative subseafloor prokaryotes. For example, Yanagibayashi *et al*. (1999) enriched a piezophilic branch of *Shewanella*, and *Moritella* species from near surface sediment from the 6292-m-deep Japan Trench, which corresponded to several sequences in the sediment 16S rRNA gene library. However, these *Bacteria* were only obtained with continuous high-pressure (65 MPa) cultivation. In contrast, only a ‘common’ marine *Pseudomonas* was obtained under atmospheric conditions (0.1 MPa). This unique study demonstrates that even using the same medium, more representative prokaryotes from deep sea, surface sediments can be obtained with constant high-pressure cultivation and sampling, and the same should apply to deep, subseafloor sediments. It may be that a combination of initial depressurization during deep sediment sampling and depressurization/repressurization even during normal high-pressure batch cultivation limits the isolation of more representative subseafloor isolates. Anaerobic conditions and processes dominant in subsurface sediments (e.g. Parkes *et al*., 2000) and prokaryotic production and consumption of gases such as, H_2_, H_2_S, CO_2_, N_2_, NH_3_ and CH_4_, might make anaerobic enrichments even more sensitive to pressure changes than the aerobic enrichments of Yanagibayashi and colleagues (1999). For example, both sulfate reduction and anaerobic oxidation of methane are strongly influenced by elevated pressure (Nauhaus *et al*., 2002; Kallmeyer and Boetius, 2004). However, these experiments were conducted with depressurized and 0.1 MPa stored samples with unknown effects on prokaryotic biodiversity and measured rates of activity.

Until now there has been no system available to sample and handle subsurface sediments under elevated pressure. In this paper we describe a new system, DeepIsoBUG, which can handle sediments under elevated pressure (max. 25 MPa) for enrichment, growth and isolation of prokaryotes at pressures up to 100 MPa. When this system is coupled with acquisition of pressurized subsurface cores using the HYACINTH drilling and core storage system (Schultheiss *et al*., 2006), and the PRESS core cutting system (Parkes *et al*., 2009), DeepIsoBUG enables the recovery and handling of cores at *in situ* pressures (up to 25 MPa) and subsequent enrichment and isolation of prokaryotes at a range of pressures, without depressurization. The results of the first use of this system are presented, here, using subsurface gas hydrate containing sediments from the Indian Continental Shelf, the Gulf of Mexico and the Cascadia Margin. The aim of this research was to investigate whether enrichment and isolation of subseafloor anaerobic prokaryotes under elevated pressure, without depressurization, would enable acquisition of different culturable prokaryotes compared with the normal procedure of using depressurized sediments and atmospheric pressure handling and isolation.

## Results

### Description of the DeepIsoBUG System

**Fig. 1:**
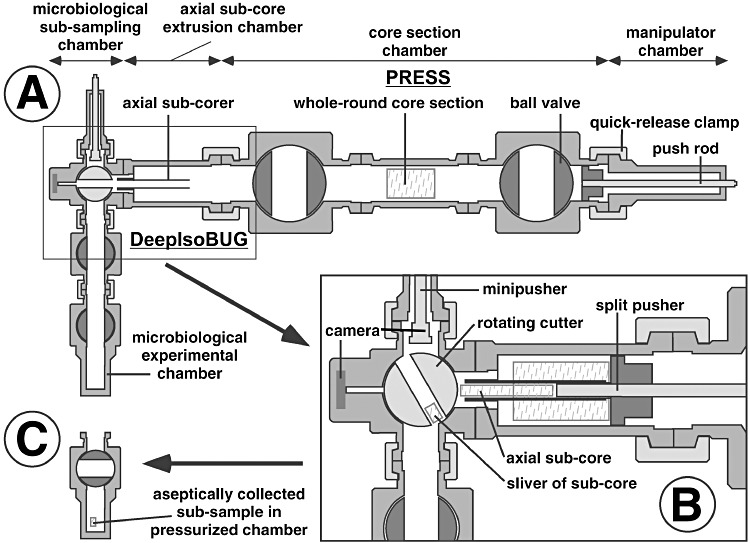
Schematic of subcoring and slicing procedure using the coupled PRESS and DeepIsoBUG system to obtain an uncontaminated central core slice for prokaryotic enrichments and other experiments. A. Complete system connected after cutting and transfer of core section. B. Blow-up showing the subcoring and slicing operation. C. Sediment slice isolated in pressure vessel (adapted from Schultheiss *et al*., 2006; not to scale).

**Fig. 2:**
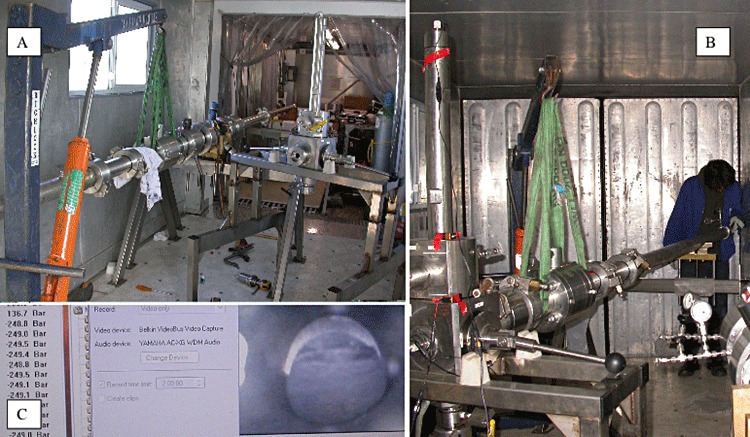
Photograph of (A) the PRESS core cutting and manipulation system, with the DeepIsoBUG core slicer separate. B. The PRESS system attached to the DeepIsoBUG core slicer ready for extrusion of a central core section, as shown schematically in (A). C. Computer screen picture of the central core within the DeepIsoBUG system ready to be sliced and transferred to a low pressure vessel, plus the pressure sensor read out (left) showing processing occurring at 13.67 MPa. The equipment is in a 40 foot, temperature-controlled container.

**Fig. 3:**
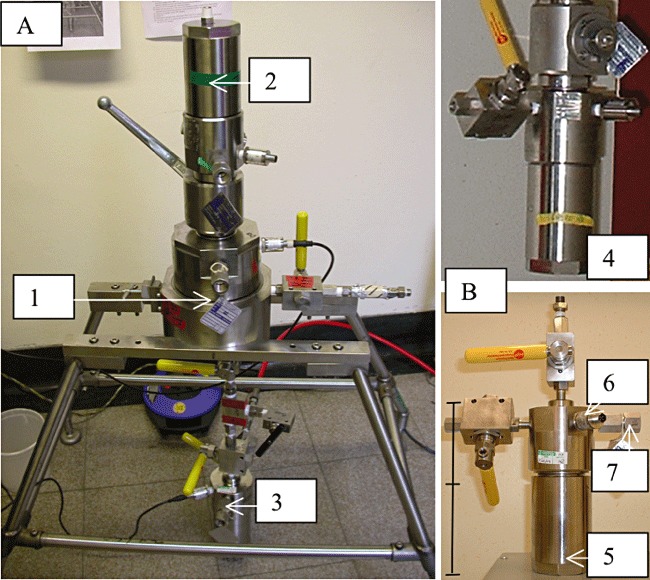
Photograph of (A) the DeepIsoBUG transfer chamber (1) enabling sediment slurry transfer under pressure (maximum 25 MPa) from low-pressure vessel (2, upside down) to a high-pressure vessel (3, maximum 100 MPa) for inoculation of medium and enrichment. B. Separate low- (4) and high-pressure (5) vessels with ball valves for transfer in and out and gas flushing; low-pressure vessel has large ball valve on top for input of sediment slice(s). Pressure sensor (6) and burst disc (7) also shown. Scale bar on the high-pressure vessel is 22 cm.

**Fig. 4:**
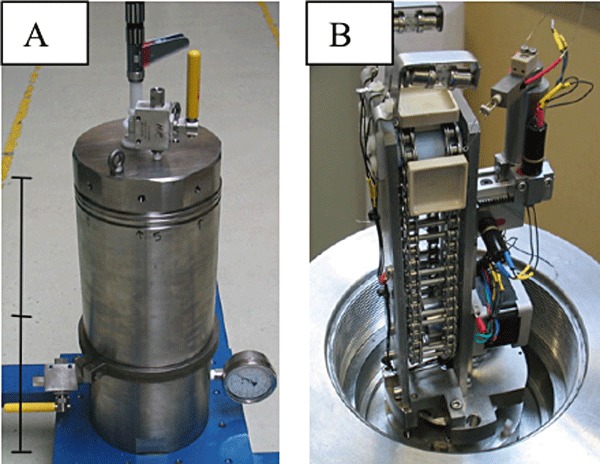
Isolation chamber outside (A) and inside (B) showing some agar plate trays on a rotating chain and inoculation and colony transfer arm. Scale bar in (A) is 64.5 cm.

The DeepIsoBUG system has several parts which are described below:

(i) A subcoring and slicing system (Figs 1 and 2), which enables a central subcore (20 mm) to be obtained from a core section and then for this to be sequentially sliced (using a manually rotated blade), with each slice being transferred (using a rotating central section) to a low pressure (max. 25 MPa) vessel with a large ball valve. Two cameras behind sapphire windows enable: (i) the subcore to be viewed as it is extruded, to ensure that sediment is present and gas voids or other disturbances are absent, and to allow for targeting of specific features, e.g. gas hydrates, sapropel layers or mineral deposits (Fig. 2C) and (ii) that the slice has been transferred to the pressure vessel (camera attached to the pushing rod, Fig. 1B). Anaerobic mineral salts medium is already in the pressure vessel (Fig. 1C), so thorough shaking produces a sediment slurry for use as inoculum.

(ii) A transfer chamber (Fig. 3A), which enables the sediment slurry to be transferred from the low-pressure vessel (5 ml slurry aliquots) to a number of high-pressure (max. 100 MPa) culture vessels containing enrichment medium. The chamber also contains a filter (∼100 μm) to stop transfer of large particles, which could damage ball valves, etc.

(iii) An isolation chamber (Fig. 4) that is based on that successfully used for isolation of deep sea, aerobic bacteria by Jannasch and colleagues (1982). It has 12 agar plates attached to an electric motor-driven chain, so individual plates can be selected (Fig. 4B). The chain drive lifts out of the isolation chamber and plates detach so that anaerobic media can be prepared and the system assembled in an anaerobic chamber and transferred (via an anaerobic bag) to the presterilized and gas-flushed chamber body. The chamber also contains eight detachable cells of up to 15 ml in volume, which enable growth of cultures in liquid medium within the chamber and also transfer in and out of the isolation chamber. Enrichments for isolation are transferred into an individual growth cell within the isolation chamber. A motorized robotic arm in the isolation chamber enables a sterile inoculation loop (Fig. 4B, electrically heated *in situ*) to be dipped into the growth cell and its contents to be streaked onto an agar plate. After incubation individual colonies can then be picked off and transferred to other cells containing sterile media for further growth. Transfer of these purified cultures out of the isolation chamber into high-pressure incubation chambers allows further subculture and physiological tests to be conducted on isolates. All manipulations in the isolation chamber are viewed through a sapphire window via an endoscope attached to a digital camera and monitor (Fig. 4A).

(iv) Pressure vessels (100 cm^3^ total volume, 80 cm^3^ medium; Fig. 3B) for enrichment and cultivation have a body and a lid, attached together with a screw thread and are each machined from a solid block of titanium. The vessels seal by a male cone in the lid and a complementary female cone in the body, plus ‘O’ ring seals. The low-pressure enrichment vessels (4 in Fig. 3B) have a small-bore ball valve (4 mm) on the lid for gas input and liquid transfer, as well as the large-bore ball valve (32 mm) for sediment slice transfer. The high-pressure, incubation vessels have just two small-bore ball valves (5 in Fig. 3B).

(v) General system conditions. The subcoring and slicing system was manufactured from stainless steel 316 while all other parts of the system were manufactured from titanium grade 5. Pressurization is hyperbaric using oxygen-free nitrogen (OFN) gas pressure. All of the systems have at least two valves (HiP, Erie, Pennsylvania, USA) to allow them to be flushed with sterile OFN, have pressure sensors and burst discs for safety (e.g. 6 and 7 in Fig. 3B). All gases are sterilized by filtration. Gas pressurization is via a series 3000 gas booster (Staffordshire Hydraulics, UK). Sterilization of the components is by autoclaving, where size permits, or steam cleaning followed by an ethanol wash and flaming, and incubation is in a constant temperature room or incubator. Pre-prepared sterile anaerobic media (e.g. Webster *et al*., 2009) are transferred into sterile pressure vessels within an anaerobic chamber. High-pressure liquid transfer between systems is achieved by a small pressure differential (∼2 MPa) between vessels. Ball valves are used to prevent shear stresses and possible cell rupture during culture transfer by pressure differential (e.g. Mitsuzaw *et al*., 2005).

**Fig. 5:**
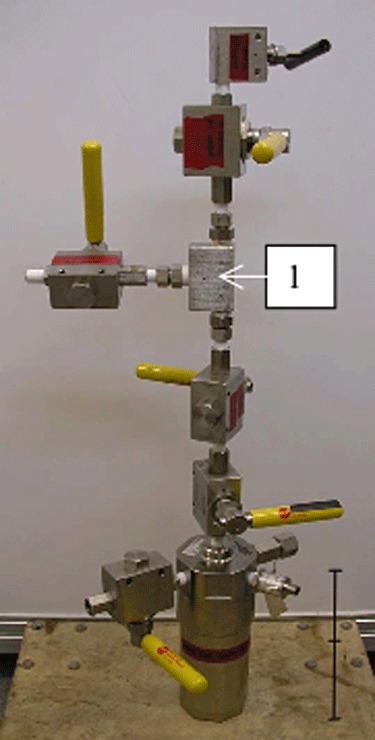
Sampling a high-pressure vessel without depressurization using a bored out ‘T’ piece (1) and a series of valves. Scale bar on the high-pressure vessel is 22 cm.

For routine high-pressure sampling of pressure vessels and subculture into vessels with fresh medium, a bored-out ‘T’-piece and a series of valves are used (Fig. 5), which enabled transfers without depressurization. All design and manufacture were to European Regulations (Pressure Equipment Directive 97/23/EC).

### Using the HYACINTH drilling system with DeepIsoBUG

The HYACINTH drilling system recovers ∼1 m long × 51–57 mm diameter cores at *in situ* pressures of up to 25 MPa. These are then transferred into core storage chambers, at the same pressure as the recovered core, and stored and transported at 2–6°C (Schultheiss *et al*., 2006). The core is removed from the storage chamber using a manipulator chamber and transferred into the PRESS system (Parkes *et al*., 2009). The PRESS cuts a section of the original core (3 to ∼10 cm), which is then coupled directly to DeepIsoBUG to begin the sediment processing described above. The HYACINTH system can be used on the Integrated Ocean Drilling Program (IODP) scientific drilling ship, Joides Resolution or a geotechnical drilling ship/rig. Contamination checks on cores obtained by IODP demonstrate that any contamination is usually restricted to the outer surface of cores (Smith *et al*., 2000), and hence, the central 20 mm subcore obtained by the PRESS/DeepIsoBUG system should be uncontaminated.

The PRESS system alone coupled to a core storage container can transfer cut core sections into commercial Parr pressure vessels (Moline, Illinois, USA) when fitted with a large ball valve. These pressure vessels can be stored and transported more easily than the larger core storage containers. The cores can then be removed using the PRESS manipulators for processing using the DeepIsoBUG system. This approach means that the DeepIsoBUG system does not always have to be transported to the site of initial high-pressure core handling.

### Tests of growth of anaerobic prokaryotes in the DeepIsoBUG pressure vessels

Before use of the high-pressure system with deep sediments the effectiveness of our anaerobic handling protocols and the ability of the pressure vessels to maintain anoxic conditions were checked with growth of the sulfate-reducing bacterium, *Desulfovibrio acrylicus*. After incubation at 25°C for 10 days between 0.1 and 44 MPa *D. acrylicus* produced significant sulfide (2.5–7 mM) and also grew under pressure after subculture using the transfer chamber.

### Cell concentrations in Gulf of Mexico, Cascadia Margin and Indian Continental Margin enrichments with elevated pressure (0.1–80 MPa)

**Fig. 6:**
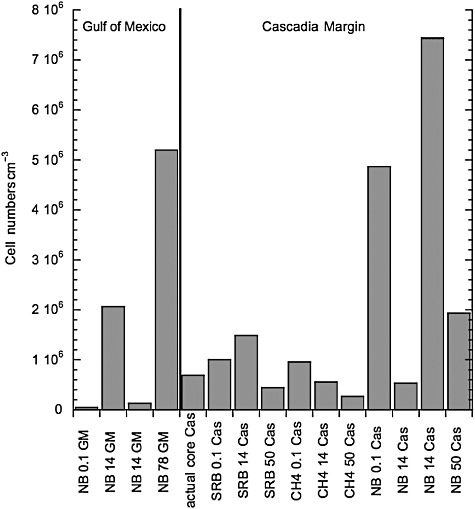
Total cell numbers in Gulf of Mexico (GM) and Cascadia Margin (Cas) anaerobic enrichments with different medium and pressures. Media = NB, SRB, methanogen (CH4, VFA medium). Numbers = pressure in MPa.

The complete HYACINTH-DeepIsoBUG system was used first in the Gulf of Mexico [Keathley Canyon, water depth ∼1300 m and 227.08 mbsf (metres below sea floor), May 2005; handling at 14 MPa]. As system protocols had to be developed and several tests conducted, only four enrichments were carried out (0.1–78 MPa), all in anaerobic nutrient broth (NB). Due to logistical problems in transporting refrigerated, high-pressure incubation vessels from Houston (Texas, USA) to Cardiff (UK), subsampling of these vessels was delayed until February 2006 (∼270 days). Despite this delay, cell numbers in the elevated pressure enrichments were always higher than those at one atmosphere (average × 48, Fig. 6). The highest cell numbers occurred at 78 MPa and in this sample there were numerous chains of cells. There was a considerable difference in the cell concentrations between the two 14 MPa enrichments, but this may reflect differences in inoculum volume, as there were problems with the transfer chamber for one of these pressure vessels.

A greater range of anaerobic media was used with the Cascadia Margin accretionary prism sediments (water and sediment depths, 1315 m and 170.5 mbsf respectively, handling at 15 MPa). Inoculated high-pressure incubation vessels were quickly transported to Cardiff University, refrigerated. After ∼14 weeks' incubation (Fig. 6) total cell counts had increased considerably and most were higher (60%) than the original sediment. For both sulfate-reducing bacteria (SRB) medium and NB, highest cell numbers occurred at 14 MPa; however, replicate results for NB at 14 MPa were again variable. In contrast, with methanogen medium highest cell numbers occurred at 0.1 MPa and decreased with increasing pressure (14 and 50 MPa were 58% and 28%, respectively, of 0.1 MPa cell numbers). Overall, growth in NB was considerably greater than for the other media (469%, despite the low count in one of the 14 MPa enrichments).

**Fig. 7:**
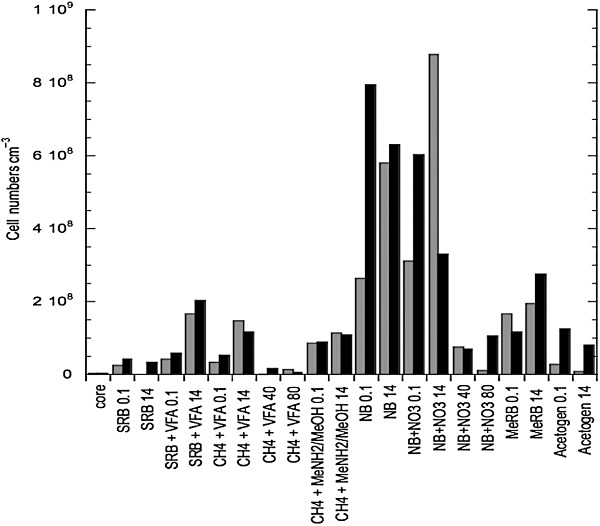
Total cell numbers in Indian Continental Margin anaerobic enrichments with different media and pressures after 6 (shaded bars) and ∼15 weeks (black bars) incubation. Media: NB (with nitrate + NO_3_), SRB (with methane or a VFA mix + VFA), methanogen (CH_4_, with acetate and formate + VFA, or methylamine and methanol + MeNH_2_/MeOH), metal reducing (MeRB) and acetogen. Numbers = pressure in MPa.

The Indian Continental Margin sediments (sediment depth 77 mbsf, water depth 1049 m, handled at 12.5 MPa) were transported in Parr pressure vessels to Cardiff laboratories for further handling. This enabled direct access to microbiological facilities and a full range of different media were inoculated (eight types) at both 0.1 and 14 MPa. In addition, methanogen with volatile fatty acid (VFA) and NB with nitrate media were also incubated at 40 and 80 MPa. After 6 weeks' incubation growth had occurred in most media and pressures (90%) when compared with the cell numbers in the original sediment (Fig. 7), and in 75% of the media maximum cell concentrations occurred at 14 MPa. However, considerable growth also occurred at 80 MPa in the two media tested (337% and 459% of sediment cell count for methanogen with VFA and NB with nitrate respectively), although surprisingly, growth in the methanogen medium at 40 MPa was lower than at 14 or 80 MPa (Fig. 7). Media containing anaerobic NB produced considerably higher cell numbers compared with enrichment with selective media (average 159% higher) and the presence of nitrate with NB gave higher cells numbers than NB alone. After ∼15 weeks' incubation (Fig. 7) there were considerable changes in the cell numbers in enrichments with, overall, decreasing numbers in the NB plus nitrate media (13% decrease) and considerable increases in numbers in other media [sulfate reducing (293%, no VFA, 124% plus VFA); NB (169%]; acetogen (568%), Fig. 7]. The rich NB media still, overall, produced the highest cell numbers and in NB plus nitrate media there was continued growth at 80 MPa. In 50% of the media maximum cell concentrations occurred at 14 MPa, but growth at 0.1 MPa now produced the highest cell numbers in NB medium. Interestingly, no nitrate removal occurred in the NB plus nitrate media, and no sulfate removal occurred in the SRB media; however, acetate production occurred in all media, including the acetogen medium (> 5000 × increase compared with uninoculated medium).

### Composition of enrichments by PCR-DGGE analysis

#### Gulf of Mexico and Cascadia Margin

*Carnobacterium*, *Clostridium*, *Pseudomonas*, *Marinilactibacillus* and other *Gammaproteobacteria* and *Firmicutes* species were present in enrichments from both the Gulf of Mexico and Cascadia Margin (data not shown). *Clostridium*, *Pseudomonas* and *Firmicutes* species occurred only in NB media at both sites, but the other genera occurred in different media and pressures (up to 80 MPa). In addition, sequences related to *Acinetobacter* species (*Gammaproteobacteria*, sulfate-reducing medium, 0.1 MPa) and *Paracoccus* species (*Alphaproteobacteria*, methanogen medium, 0.1 MPa) occurred in Cascadia Margin enrichments only, and *Marinobacter*-(*Gammaproteobacteria*) and *Acidovorax*-like sequences (*Betaproteobacteria*, both NB, 0.1 MPa) occurred in Gulf of Mexico enrichments only, but, overall, these were not dominant. The bacterial community composition of enrichments from both sites was largely stable, but no *Archaea* were detected.

#### Indian Continental Margin

**Fig. 8:**
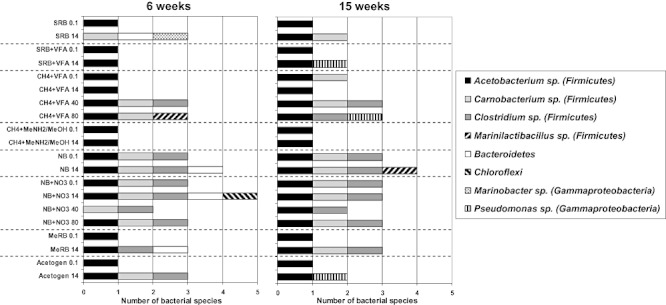
Bacterial composition (PCR-DGGE) of Indian Continental Margin anaerobic enrichments with different media and pressures after 6 and ∼15 weeks incubation. Media as for Fig. 7. Numbers = pressure in MPa.

The community composition of Indian Continental Margin enrichments ∼6 weeks after inoculation had considerable similarities to the Gulf of Mexico and Cascadia Margin enrichments, with *Carnobacterium* (23%), *Clostridium* (21%), *Marinilactibacillus* (2%) and *Marinobacter* (2%) species present (Fig. 8). In addition, *Acetobacterium* (40%) and *Bacteroidetes* (9%) were also present. There was little variation in composition between different media and pressures with both *Carnobacterium* and *Acetobacterium* species being particularly widespread. There was a tendency for *Clostridium* species to be more consistently present in the rich NB media. One *Chloroflexi* sequence with 100% sequence similarity to a clone [clone IODP 1320B92.20 (Accession Number AB433090)] previously retrieved from Gulf of Mexico (IODP Expedition 308 site 1319) sediments was detected in the NB plus nitrate enrichment at 14 MPa. This, however, disappeared along with *Bacteroidetes* and *Marinobacter* species, after ∼15 weeks' incubation (Fig. 8). In addition, a few *Pseudomonas* species appeared (7%), all at elevated pressure, including 80 MPa. However, similar proportions of the dominant bacterial types (*Carnobacterium* 21%, *Acetobacterium* 45%, *Clostridium* 24%) show that the community was relatively stable. Hence this bacterial composition was maintained through two subcultures, without the pressure vessels being depressurized. Again no *Archaea* were detected.

A subset of the enrichments were selected for pure culture isolation based on targeting first the full diversity present, second enrichments from the full pressure range and third those enrichments with single or fewest bacterial types present.

### Prokaryotic diversity of original Indian hydrate sediment

Despite the use of nested PCR-DGGE, only limited bacterial 16S rRNA gene sequence data were obtained from the 77 mbsf sediments used for the high-pressure enrichments (data not shown). Replicate DGGE profiles and sequencing showed only two bands belonging to members of the phyla *Firmicutes* and *Actinobacteria*[100% sequence similarity to *Lactococcus lactis* (Accession Number EF694031) and 99% to *Rhodococcus erythropolis* (Accession Number EU004423) respectively]. However, these bacterial sequences were not closely related to our enrichments or isolates. Repeated nested PCR amplification of sediment DNA with a number of archaeal primer sets failed to detect any *Archaea* in these sediments.

### Isolation of pure cultures from Indian gas hydrate sediment enrichments

To our surprise all selected enrichments grew at atmospheric pressure despite previous constant cultivation at up to 80 MPa for ∼560 days; therefore, isolation was conducted under atmospheric pressure. All cultures grew on NB agar plates either aerobically or anaerobically. Purification, however, required isolation on more defined YPG or YPGL media, except for the *Acetobacterium* that required anaerobic lactate medium and agar shake tube isolation. In all, six different bacterial genera were isolated in pure culture (Table 1) and these represented 70% and 100% of the DGGE sequences detected after ∼6 and 15 weeks' enrichment respectively. Different enrichment media and pressures did not always result in isolation of different genera (Table 1, supported by similar results with parallel hydrostatic pressure incubations, G. Sellek, G. Webster, H. Sass, A. J. Weightman and R. J. Parkes, unpubl. results).

**Table 1:** Isolates obtained from Indian Continental Margin gas hydrate sediment anaerobic enrichments at different pressures, percentage similarity of isolates to DGGE bands from enrichments (or in parenthesis, representative DGSE bands in other enrichments) and closest cultured strains

Taxonomic group	Isolate	Enrichment source	Pressure (MPa)	Metabolism	% Similarity to DGGE band in enrichment	Nearest cultured relative using RDP sequence match (accession #)	% Similarity
*Gamma proteobacteria*	*Pseudomonas* sp.	CH_4_ + VFA	0.1	Aerobic	(93)	Arctic seawater bacterium strain R7078 (AJ293824) *Pseudomonas* sp. strain 1_C16_29 (EF540457)	99 98
*Firmicutes*	*Carnobacterium* sp. 1	NB + NO_3_	40	Facultative anaerobe	99	*Carnobacterium pleistocenium*^T^ strain FTR1 (AF450136)	98
	*Carnobacterium* sp. 2	NB + NO_3_	80	Facultative anaerobe	99	*Carnobacterium pleistocenium*^T^ strain FTR1 (AF450136)	96
	*Marinilactibacillus* sp. 1	CH_4_ + VFA	14	Facultative anaerobe	(100)	*Marinilactibacillus piezotolerans* strain JCM 12337 (AB294178)	99
	*Marinilactibacillus* sp. 2	NB + NO_3_	80	Facultative anaerobe	(100)	*Marinilactibacillus piezotolerans* strain JCM 12337 (AB294178)	99
	*Acetobacterium* sp.	NB + NO_3_	40	Anaerobe	(100)	*Acetobacterium carbinolicum*^T^ DSM 2925 (X96956)	99
	*Clostridium* sp. 1	NB + NO_3_	80	Anaerobe	100	Bacterium strain UXO5-23 (DQ522110) *Clostridium scatologenes* DSM 757 (DQ911270)	99 93
*Bacteroidetes*	*Bacteroidetes* sp.	Acetogen	14	Facultative anaerobe	(99)	Bacterium strain JAM-BA0302 (AB362263) *Prolixibacter bellariavorans* strain F2 (AY918928)	99 89

## Discussion

This study has several unique aspects: the direct enrichment of subseafloor prokaryotes under elevated pressure; enrichment of sedimentary, anaerobic prokaryotes without depressurization; and high-pressure cultivation from subsurface gas hydrate sediments. Results clearly show the ability of many subseafloor prokaryotes from gas hydrate-containing sites to grow up to at least 80 MPa in a variety of anaerobic media. This is consistent with the widespread occurrence of subseafloor prokaryotes (Parkes *et al*., 2000), although growth at 80 MPa is a much higher pressure than has previously been observed for subseafloor prokaryotes [e.g. up to ∼40 MPa (Bale *et al*., 1997) and ∼30 MPa (Toffin *et al*., 2005)]. As 70% of the ocean is at 38 MPa or above and the average depth of sediments is 500 m, growth at 80 MPa would enable the majority of the subseafloor environment to be populated by prokaryotes. However, none of the enriched prokaryotes are obligate piezophiles as there was little difference in community composition with incubation pressure (Fig. 8, Indian hydrate samples) and almost all the enriched bacteria grew at atmospheric pressure during isolation. This is despite the higher incubation pressures used being in the range for extreme and obligate piezophiles (Kato *et al*., 1998). Hence, it appears that the community that developed in our high-pressure incubations were piezotolerant.

These results are in agreement with the dominance of piezotolerant, aerobic bacterial isolates from 4000 m deep seawater samples, obtained without depressurization (Jannasch *et al*., 1982). In this study it was considered that piezotolerant bacteria were recent arrivals from shallow waters, reaching deep water on the considerable particle flux that occurs. However, as our sediment samples were from 77 to 227 m below the sediment surface they certainly do not contain recent arrivals from near surface seawater. In addition, maximum cell concentrations during enrichment often occurred at 14 MPa, close to *in situ* pressures (11–15 MPa), demonstrating some piezophilic characteristics. Also there was growth at 50 to ∼80 MPa in enrichments from all three of our sites, much higher than *in situ* pressures, which might reflect upward flux of deeper adapted prokaryotes with fluids and gases, potentially including deep crustal fluids (e.g. Cowen *et al*., 2003; Edwards *et al*., 2005). Obligate piezophiles have been obtained from surface sediments from the deepest ocean Trench (Challenger Deep, 10 898 m, Kato *et al*., 1998) and perhaps our samples are not deep enough to obtain these extreme piezophiles (total depth 1126–1527 m). Insufficiently deep samples may also explain why there was little difference in community composition with incubation pressure with the Indian gas hydrate samples and that decompression prior to high-pressure cultivation had seemingly limited effect on composition of the enriched community (composition of hydrostatic enrichments being similar, G. Sellek, G. Webster, H. Sass, A. J. Weightman and R. J. Parkes, unpubl. results). The presence of only piezotolerant *Bacteria* meant that it was unnecessary to use the high-pressure isolation chamber as 0.1 MPa isolation obtained all dominant bacterial types present after initial subculture of enrichments. Except for the *Pseudomonas* (93%, Table 1) isolates were 99–100% similar to DGGE band sequences in the enrichments.

It is intriguing that no *Archaea* were enriched, as these have been suggested to be the dominant prokaryotes in subseafloor sediments (Lipp *et al*., 2008), and with their robust cell membrane and tolerance of extreme conditions (Valentine, 2007), they may have been considered to be favoured under high-pressure cultivation. In addition, either their proposed heterotrophic (Biddle *et al*., 2006) or known methane-linked metabolism [methanogenesis (acetate and methylamine plus methanol media) and anaerobic oxidation of methane, SRB medium with methane)] should have resulted in preferential enrichment with some of the selective media used. However, none of the media used seemed to make a significant difference to the enrichment community obtained (Fig. 8) and none of the selective media resulted in isolation of the targeted group. However, targeted acetogens were isolated from non-selective organic rich medium (Table 1) and were enriched in a range of different media, including acetogen medium (Fig. 8). Perhaps all the media used discriminated against the low-energy adaptation, recently suggested to be a characteristic of *Archaea* (Valentine, 2007). Conversely, the absence of archaeal enrichments may reflect their low *in situ* cell numbers in deep sediments, as suggested by some molecular genetic studies (Schippers *et al*., 2005), including those considering methanogens (Fry *et al*., 2008). There was some selective enrichment of *Carnobacterium* and *Clostridium* species in the rich NB medium, but these species also occurred, although not as consistently, in other media (Fig. 8). Perhaps this is because these and other common bacterial genera enriched have relatively broad metabolisms, especially when in mixed culture, as in these enrichments. Indeed, it proved difficult to obtain pure culture isolates from several of the enrichments, taking up to seven colony isolation steps to obtain pure cultures. In addition, the dominant bacterial composition of the enrichments was maintained for at least two high-pressure subcultures, showing both the stability of the enrichments and also the suitability of our high-pressure transfer procedures.

Unfortunately, only limited 16S rRNA gene sequence data were obtained directly from the Indian gas hydrate sediments (*Firmicutes* and *Actinobacteria*), despite the use of sensitive nested PCR. These sediment sequences were not closely related to bacterial sequences in enrichments or isolates. This probably reflected the relatively low total cell numbers (3.3 × 10^6^ cm^−3^) in the original sediment and subsequent low concentrations of extractable DNA (Webster *et al*., 2003; Fry *et al*., 2008). However, the dominant bacterial groups in other subseafloor sediments detected by molecular genetic techniques include *Gammaproteobacteria*, *Chloroflexi* and members of the JS1 candidate division (Fry *et al*., 2008). Only the former has close cultured relatives, including pseudomonads, which were isolated here. In addition, *Carnobacterium*- and *Clostridium*-like sequences have been detected in a number of subsurface environments, including deep marine sediments from the Nankai Trough, subsurface paleosols and a deep gas storage aquifer (Chandler *et al*., 1998; Newberry *et al*., 2004; Basso *et al*., 2009), and *Carnobacterium* and *Clostridium* species were isolated from the Indian subsurface sediments. Also, *Acetobacterium carbinolicum*, which was isolated from our Indian hydrate samples, was detected in the deep gas storage aquifer (Basso *et al*., 2009).

In subsurface gas hydrate sediments *Gammaproteobacteria* are also present but not dominant, along with *Bacteroidetes* and *Firmicutes* (Inagaki *et al*. 2006), which were also isolated from Indian gas hydrate samples (Table 1). *Bacteroidetes*-related sequences have also previously been detected at 234 mbsf in Cascadia Margin subsurface hydrate deposits (Marchesi *et al*., 2001) and *Pseudomonas* and *Bacteroidetes* sequences detected in Nankai Trough deep gas hydrate sediments (Reed *et al*., 2002). *Firmicutes* have similarly been found in a number of gas hydrate sediments (e.g. Lanoil *et al*., 2001), including *Clostridium*-like sequences in gas hydrate mounds from the Gulf of Mexico (Mills *et al*., 2003).

The dominant isolates obtained from our Indian gas hydrate samples were different from those from some other deep sediments, such as Equatorial Pacific Ocean and Peru Margin down to 420 m (Batzke *et al*., 2007), which were *Bacillus* and *Rhizobium*, although *Gammaproteobacteria*, *Bacteroidetes* and other *Firmicutes* were also isolated. Interestingly, as found here, Batzke and colleagues (2007) did not isolate any *Archaea*; their isolate distribution did not correlate with medium; and although their enrichments were conducted under anoxic conditions, isolates were mostly facultative anaerobes. In our study with Indian hydrate samples the obligate anaerobic *Acetobacterium* and *Clostridium* species were isolated (Table 1). Similar bacterial groups were found in deep Peru Margin sediments (near surface to 178 mbsf) by Biddle and colleagues (2005), although, interestingly neither *Bacillus* or *Rhizobium* species were detected. Enrichments from subsurface sediments from Nankai Trough (4.15 m, Toffin *et al*., 2004) are most similar to our Indian hydrate enrichments, as these contained *Carnobacterium*, *Acetobacterium*, *Clostridium* and *Pseudomonas* species, in addition to *Bacillus*, *Spirochaeta* and *Desulfofrigus*. However, Nankai Trough pure culture isolates were restricted to *Acetobacterium* and *Marinilactibacillus* sp., in contrast to our broader range of isolates (Table 1).

Indian gas hydrate sediment isolates (Table 1) had between 89% and 99% 16S rRNA gene sequence similarity to previously isolated strains, including some from other deep sediments (*Marinilactibacillus*, Toffin *et al*., 2005; *Bacteroidetes*, Kobayashi *et al*., 2008). Some close relatives were psycrophiles/tolerant and/or from permanently cold environments (*Pseudomonas*, Polar Seawater, Mergaert *et al*., 2001; *Carnobacterium*, Subsurface Pleistocene ice, Pikuta *et al*., 2005). If our isolates have similar physiological characteristics this would have helped them to grow under the high-pressure enrichment conditions, as many psychrophilic adaptations can also facilitate growth under pressure (Lauro *et al*., 2007). The *Clostridium* species was most closely related to a novel group of *Firmicutes* isolated from shallow marine sediments contaminated with explosives (Zhao *et al*., 2007). Interestingly, the closest described pure culture to this isolate is *Clostridium scatologenes* (93%), which has recently been found to be capable of acetogenic activity (Kusel *et al*., 2000). This together with the presence of the *Acetobacterium* species (99% similarity to *A. carbinolicum*, Eichler and Schink, 1984) would explain the increased acetate that occurred in the enrichments. Elevated pore water acetate concentrations also occurred *in situ* in the Indian gas hydrate sediment sample (∼1650 μM) and even higher concentrations have been detected at other deep subsurface gas hydrate sites (e.g. Blake Ridge, Atlantic Ocean, Wellsbury *et al*., 1997).

Similar groups of facultative anaerobic *Bacteria* to those obtained from the Indian gas hydrate sediments were also enriched under elevated pressure and isolated from the two other gas hydrate sites, Cascadia Margin and Gulf of Mexico (G. Sellek, G. Webster, D. Martin, H. Sass, A. J. Weightman and R. J. Parkes, unpubl. results), including positive enrichments at 80 MPa with Gulf of Mexico sediments (Cascadia Margin enrichments maximum pressure was 50 MPa). This demonstrates the presence of a common group of culturable, piezotolerant *Bacteria* in subsurface gas hydrate-containing sediments, most of which can grow up to at least 80 MPa and grow to maximum cell densities during initial enrichment close to *in situ* pressures (∼14 MPa, possibly higher in Gulf of Mexico). This includes *Carnobacterium* species that have recently been shown to contain hyperpiezophilic strains (optimal growth pressure > 60 MPa, Lauro *et al*., 2007) and the sister taxon *Marinilactobacillus* that contains a psychropiezotolerant isolate (Toffin *et al*., 2005).

This is the first report of the isolation of *Carnobacterium*, *Marinilactobacillus*, *Acetobacteium*, *Clostridium* and *Bacteroidetes* species from deep, subseafloor, gas hydrate deposits. In addition, growth of *Bacteria* up to 80 MPa is a much higher pressure than has previously been demonstrated for subseafloor prokaryotes and is consistent with the large estimated prokaryotic biomass in deep, subseafloor sediments (Whitman *et al*., 1998).

## Experimental procedures

### Site descriptions and sediment core processing

#### Gulf of Mexico

Sediment samples were obtained during the Gulf of Mexico Hydrates Joint Industry Project. The sediment samples were at 227.08 mbsf (core KC151-311P, obtained with a Fugro Pressure Corer) from the Keathley Canyon site (approximately 400 km SE of Galveston, Texas) in a water depth of approximately 1300 m. Down hole logging indicated the presence of gas hydrates from 220 to 300 mbsf, but no physical hydrate was recovered. High methane concentrations (e.g. 10 mM at 10 mbsf) were ^13^C-depleted, indicating a predominantly microbial origin. *In situ* temperature was estimated to be between 6°C and 9°C (Claypool, 2006). The core was kept at 14 MPa in seawater until processed on 29 and 30 May 2005 at Houston, Texas.

#### Cascadia Margin

Subsurface sediment samples from Cascadia Margin (west of Vancouver Island, north-east Pacific Ocean) were obtained during IODP Expedition 311. A high-pressure core from 170.5 to 171.5 mbsf (12E) in Hole U1327D at a water depth of 1315.4 m was collected (HYACE Rotary Corer) then stored at 13.5 MPa. The occurrence of gas hydrates above a bottom simulating reflector at ∼225 mbsf was indicated by a range of techniques (Expedition, 311 Scientists, 2005). Methane was considered to be of microbial origin and there was a temperature gradient of ∼6°C/100 m. The core was processed on the 9 November 2005 under constant high pressure (15 MPa) and temperature (∼4–6°C) at the Geological Survey of Canada – Pacific Trailer Park at Sidney, BC, Canada.

#### Indian Continental Margin

Sediment from 77 mbsf at Site 21 (water depth 1049 m) in the Krishna-Godavari Basin (Bay of Bengal off the Indian East Coast) on the Indian Continental Margin was obtained from Expedition 01 of the Natural Gas Hydrate Program (India). The sediment contained considerable gas hydrate concentrations (up to 15% of the formation) in thin veins. Pore waters (G. Webster, E. G. Roussel and R. J. Parkes, unpubl. results) had elevated concentrations of the VFA acetate (∼1650 μM) and depleted sulfate concentrations (5 mM), which would have facilitated *in situ* microbial methane production. The PRESS system was used to transfer cut core sections into Parr Pressure Vessels at 12 MPa, which were stored and transported to the UK at 4°C. Hydrate layers were present in a frozen subsample of the sediment used for molecular genetic analysis. Further core processing took place in Cardiff under constant pressure (14 MPa) and ∼15°C.

All the enrichments from these sediments were incubated at 10°C.

### Culture media

Eight different culture media were used with Indian gas hydrate sediments and a subset of these was used with the other two sites. Basal media composition was artificial seawater (ASW; Süß*et al*., 2004) with the following concentrations: NaCl (24.3 g l^−1^), MgCl_2_·6H_2_O (10 g l^−1^), CaCl_2_·2H_2_O (1.5 g l^−1^), KCl (0.66 g l^−1^), Na_2_SO_4_ (1.42 g l^−1^) plus the following added from liquid stock solutions: SL10 trace elements (1 ml l^−1^) and Selenite-Tungstate solution (0.2 ml l^−1^, Widdel and Bak, 1992) and 1 ml l^−1^ of each of the following: KBr (0.84 mM), H_3_BO_3_ (0.4 mM), SrCl_2_ (0.15 mM), NH_4_Cl (0.4 mM), KH_2_PO_4_ (0.04 mM) and NaF (0.07 mM). The medium was autoclaved at 121°C for at least 60 min. The medium was cooled under an atmosphere of N_2_ : CO_2_ (80%:20% v : v; 5 kPa). When cold, 30 ml l^−1^ NaHCO_3_ (1 M) and 10 ml l^−1^ of a vitamins solution (Balch *et al*., 1979) were added along with any additional media components (see below). All media were pH 7.4 ± 0.2, and did not require further pH adjustment. Anaerobic medium was reduced with 0.5 mM FeSO_4_ or FeCl_2_ and 1.5 mM Na_2_S except for SRB medium, which was reduced by the addition of sodium dithionite. Un-supplemented basal medium was used for producing initial sediment slurries from core slices.

General heterotrophic medium (NB) was based on NB (Merck Nutrient Broth No. 2) with final concentrations of 10 g l^−1^ meat extract and 10 g l^−1^ peptone. Salts were added to produce the same salinity as the ASW. All other media supplements (vitamins, FeS, bicarbonate) were added as described above. NB was used with (NB + NO_3_) and without (NB) 3 mM sodium nitrate. Medium for metal-reducing bacteria (MeRB): the basal medium was modified to provide the following concentrations: 3 mM sodium lactate, 5 mM sodium acetate, 2 mM sodium formate, 2 mM sodium propionate, 1 mM sodium butyrate plus 20 mM amorphous iron hydroxide and 10 mM manganese oxides (Köpke *et al*., 2005) as electron acceptors. Medium for heterotrophic SRB (SRB + VFA), additions to basal medium produced the following concentrations: sodium sulfate (30 mM), sodium lactate (3 mM), sodium acetate (5 mM), sodium formate (2 mM), sodium propionate (2 mM) and sodium butyrate (1 mM). Resazurin was also added (0.25 mg l^−1^) as a redox indicator. The medium was reduced by adding a small amount of sodium dithionite until the resazurin turned colourless. SRB medium for enrichment of anaerobic oxidation of methane prokaryotes (SRB) was based on the FeS-reduced ASW. The headspace was flushed and partly pressurized with CH_4_ : argon (95%:5% v : v; 7.5 MPa) and then fully pressurized to 14 MPa with OFN. Methanogen media: two types of methanogen media were prepared: CH_4_ + VFA contained 10 mM acetate and 5 mM formate, whereas the other, CH4 + MeNH_2_/MeOH, targeting methylotrophic methanogens contained 5 mM methylamine and 5 mM methanol. Acetogen medium: consisted of basal medium plus 2 mM vanillin and 10 mM ethanol.

Isolation for most bacteria was by repeated streaking and single-colony transfer on solid aerobic or anaerobic media at 25°C. Pure cultures were obtained by using more defined oxic or anoxic diluted yeast extract-peptone media (Süß*et al*., 2004) containing yeast extract (0.03 g l^−1^), peptone (0.06 g l^−1^), glucose (5 mM) and sodium lactate (5 mM) (YPGL) or only glucose (YPG) made with ASW. This medium was also used for strain maintenance. Isolation of *Acetobacterium* sp. was in anoxic ASW and sodium lactate (20 mM) media with repeated agar shake tube isolation. Purity was based on homogeneous cell type in phase contrast microscopy, and by PCR-DGGE and sequencing of 16S rRNA genes of cultures grown in a range of media.

### Analysis of cultures

Cell counts were obtained by Acridine Orange Direct Counting based on that described by Fry (1988). Samples for Acridine Orange Direct Counting were fixed in 2% (v/v) formaldehyde. Subsequently, a subsample, between 5 and 50 μl, was stained (0.1% (w/v) acridine orange) and then counted on a black polycarbonate membrane filter (0.2 μm) using an epifluorescence microscope. Average confidence limits of direct counts were ∼40%. Cell morphology was determined by phase contrast microscopy. Culture supernatants were measured for VFAs, sulfate and nitrate on a Dionex ICS-2000 Ion Chromatography System equipped with an AS50 autosampler (Dionex UK) as described by Webster and colleagues (2009).

### Molecular genetic analysis

#### DNA extraction from elevated pressure enrichments and bacterial isolates and PCR amplification of 16S rRNA genes

Cells were harvested from all high-pressure enrichment cultures by centrifugation (5 ml) and DNA extracted from cell pellets directly using the FastDNA SPIN Kit for Soil (MP Biomedicals) as previously described (Webster *et al*., 2003). Cells from all pure culture isolates were also harvested by centrifugation (1–5 ml depending on cell density) and DNA extracted using the Nexttec DNA Isolation System for Bacteria (Nexttec Biotechnologie). Bacterial 16S rRNA gene sequences were amplified by PCR from extracted DNA using the primer combinations 27F-907R or 27F-1492R respectively (Webster *et al*., 2009). PCR products were then analysed by DGGE (enrichments and pure cultures) or cleaned (pure cultures) using PCR clean-up centrifugal filter devices (Millipore Corporation) and sequenced using an ABI PRISM 3130xl Genetic analyser (Applied Biosystems).

Closest relatives to obtained sequences were identified by NCBI nucleotide–nucleotide blast (http://www.ncbi.nlm.nih.gov/) and phylogenetic analysis carried out using neighbor-joining with the Jukes and Cantor correction algorithm in MEGA version 4.0 (Tamura *et al*., 2007). All 16S rRNA gene sequences for pure cultures isolated in this study have been submitted to the EMBL database under accession numbers FN397989–FN397996.

#### DGGE analysis of high-pressure enrichment cultures and bacterial isolates

All bacterial 16S rRNA gene PCR products were re-amplified by nested PCR with primers 357FGC-518R (Webster *et al*., 2009). Nested PCR products were then analysed by DGGE as described by Webster and colleagues (2003). PCR products (approximately 100 ng of each PCR product) were separated using a DCode Universal Mutation Detection System (Bio-Rad Laboratories) and 1 mm thick (16 cm × 16 cm glass plates) 8% (w/v) polyacrylamide gels with a gradient of denaturant between 30% and 60%. Gels were poured with a 50 ml volume Gradient Mixer (Fisher Scientific) and prepared with 1× TAE buffer. Electrophoresis was carried out at 200 V for 5 h (with an initial 10 min at 80 V) at 60°C. Polyacrylamide gels were stained with SYBRGold nucleic acid gel stain (Molecular Probes) for 30 min and viewed under UV. Gel images were captured with a Gene Genius Bio Imaging System (Syngene). Representative DGGE bands of all unique banding positions from each sediment enrichment were excised (25/35, 23/25 and 49/86 of all DGGE bands from Gulf of Mexico, Cascadia Margin and Indian Continental Margin enrichments respectively), sequenced (O'Sullivan *et al*., 2008) and identified by NCBI nucleotide–nucleotide blast (http://www.ncbi.nlm.nih.gov/).

## References

[b10] Abe F, Horikoshi K (2001). The biotechnological potential of piezophiles. Trends Biotechnol.

[b1001] Balch WE, Fox GE, Magrum LJ, Woese CR, Rolfe RS (1979). Methanogens: reevaluation of a unique biological group. Microbiol Rev.

[b21] Bale SJ, Goodman K, Rochelle PA, Marchesi JR, Fry JC, Weightman AJ, Parkes RJ (1997). *Desulfovibrio profundus* sp nov, a novel barophilic sulfate- reducing bacterium from deep sediment layers in the Japan Sea. Int J Syst Bacteriol.

[b32] Basso O, Lascourreges JF, Le Borgne F, Le Goff C, Magot M (2009). Characterization by culture and molecular analysis of the microbial diversity of a deep subsurface gas storage aquifer. Res Microbiol.

[b8] Batzke A, Engelen B, Sass H, Cypionka H (2007). Phylogenetic and physiological diversity of cultured deep-biosphere bacteria from equatorial Pacific Ocean and Peru Margin sediments. Geomicrobiol J.

[b38] Biddle JF, House CH, Brenchley JE (2005). Microbial stratification in deeply buried marine sediment reflects changes in sulfate/methane profiles. Geobiology.

[b28] Biddle JF, Lipp JS, Lever MA, Lloyd KG, Sorensen KB, Anderson R (2006). Heterotrophic *Archaea* dominate sedimentary subsurface ecosystems off Peru. Proc Natl Acad Sci USA.

[b30] Chandler DP, Brockman FJ, Bailey TJ, Fredrickson JK (1998). Phylogenetic diversity of *Archaea* and *Bacteria* in a deep subsurface paleosol. Microb Ecol.

[b1002] Claypool GE (2006). http://www.netl.doe.gov/technologies/oil-gas/publications/Hydrates/reports/GOMJIPCruise05.pdf.

[b24] Cowen JP, Giovannoni SJ, Kenig F, Johnson HP, Butterfield D, Rappe MS (2003). Fluids from aging ocean crust that support microbial life. Science.

[b4] D'Hondt S, Jorgensen BB, Miller DJ, Batzke A, Blake R, Cragg BA (2004). Distributions of microbial activities in deep subseafloor sediments. Science.

[b25] Edwards KJ, Bach W, McCollom TM (2005). Geomicrobiology in oceanography: microbe–mineral interactions at and below the seafloor. Trends Microbiol.

[b44] Eichler B, Schink B (1984). Oxidation of primary aliphatic-alcohols by *Acetobacterium carbinolicum* sp. nov., a homoacetogenic anaerobe. Arch Microbiol.

[b47] Expedition, 311 Scientists (2005). 10.2204/iodp.pr.311.2005.

[b1003] Fry JC, Austin B (1988). Determination of biomass. Methods in Aquatic Bacteriology.

[b9] Fry JC, Parkes RJ, Cragg BA, Weightman AJ, Webster G (2008). Prokaryotic biodiversity and activity in the deep subseafloor biosphere. FEMS Microbiol Ecol.

[b33] Inagaki F, Nunoura T, Nakagawa S, Teske A, Lever M, Lauer A (2006). Biogeographical distribution and diversity of microbes in methane hydrate-bearing deep marine sediments, on the Pacific Ocean Margin. Proc Natl Acad Sci USA.

[b17] Jannasch HW, Wirsen CO, Taylor CD (1982). Deep-sea bacteria – isolation in the absence of decompression. Science.

[b1] Jorgensen BB, Schulz HD, Zabel M (2000). *Bacteria* and marine biogeochemistry. Marine Geochemistry.

[b14] Kallmeyer J, Boetius A (2004). Effects of temperature and pressure on sulfate reduction and anaerobic oxidation of methane in hydrothermal sediments of Guaymas Basin. Appl Environ Microbiol.

[b23] Kato C, Li L, Nogi Y, Nakamura Y, Tamaoka J, Horikoshi K (1998). Extremely barophilic bacteria isolated from the Mariana Trench, Challenger Deep, at a depth of 11,000 meters. Appl Environ Microbiol.

[b1004] Kobayashi T, Koide O, Mori K, Shimamura S, Matsuura T, Miura T (2008). Phylogenetic and enzymatic diversity of deep subseafloor aerobic microorganisms in organics- and methane-rich sediments off Shimokita Peninsula. Extremophiles.

[b50] Köpke B, Wilms R, Engelen B, Cypionka H, Sass H (2005). Microbial diversity in coastal subsurface sediments – a cultivation approach using various electron acceptors and substrate gradients. Appl Environ Microbiol.

[b43] Kusel K, Dorsch T, Acker G, Stackebrandt E, Drake HL (2000). *Clostridium scatologenes* strain SL1 isolated as an acetogenic bacterium from acidic sediments. Int J Syst Evol Microbiol.

[b36] Lanoil BD, Sassen R, La Duc MT, Sweet ST, Nealson KH (2001). *Bacteria* and *Archaea* physically associated with Gulf of Mexico gas hydrates. Appl Environ Microbiol.

[b42] Lauro F, Chastain R, Blankenship L, Yayanos A, Bartlett D (2007). The Unique 16S rRNA genes of piezophiles reflect both phylogeny and adaptation. Appl Environ Microbiol.

[b26] Lipp JS, Morono Y, Inagaki F, Hinrichs KU (2008). Significant contribution of *Archaea* to extant biomass in marine subsurface sediments. Nature.

[b34] Marchesi JR, Weightman AJ, Cragg BA, Parkes RJ, Fry JC (2001). Methanogen and bacterial diversity and distribution in deep gas hydrate sediments from the Cascadia Margin as revealed by 16S rRNA molecular analysis. FEMS Microbiol Ecol.

[b1005] Mergaert J, Verhelst A, Cnockaert MC, Tan TL, Swings J (2001). Characterization of facultative oligotrophic bacteria from polar seas by analysis of their fatty acids and 16S rDNA sequences. Syst Appl Microbiol.

[b37] Mills HJ, Hodges C, Wilson K, MacDonald IR, Sobecky PA (2003). Microbial diversity in sediments associated with surface- breaching gas hydrate mounds in the Gulf of Mexico. FEMS Microbiol Ecol.

[b19] Mitsuzawa S, Deguchi S, Takai K, Tsujii K, Horikoshi K (2005). Flow-type apparatus for studying thermotolerance of hyperthermophiles under conditions simulating hydrothermal vent circulation. Deep Sea Res Part I Oceanogr Res Pap.

[b13] Nauhaus K, Boetius A, Kruger M, Widdel F (2002). In vitro demonstration of anaerobic oxidation of methane coupled to sulphate reduction in sediment from a marine gas hydrate area. Environ Microbiol.

[b31] Newberry CJ, Webster G, Cragg BA, Parkes RJ, Weightman AJ, Fry JC (2004). Diversity of prokaryotes and methanogenesis in deep subsurface sediments from the Nankai Trough, Ocean Drilling Program Leg 190. Environ Microbiol.

[b52] O'Sullivan LA, Webster G, Fry JC, Parkes RJ, Weightman AJ (2008). Modified linker-PCR primers facilitate complete sequencing of DGGE bands. J Microbiol Methods.

[b3] Parkes RJ, Cragg BA, Bale SJ, Getliff JM, Goodman K, Rochelle PA (1994). Deep bacterial biosphere in Pacific-Ocean sediments. Nature.

[b12] Parkes RJ, Cragg BA, Wellsbury P (2000). Recent studies on bacterial populations and processes in subseafloor sediments: a review. Hydrogeol J.

[b7] Parkes RJ, Webster G, Cragg BA, Weightman AJ, Newberry CJ, Ferdelman TG (2005). Deep sub-seafloor prokaryotes stimulated at interfaces over geological time. Nature.

[b16] Parkes RJ, Amann H, Holland M, Martin D, Schultheiss PJ, Anders E, Collett T, Johnson A, Knapp C, Boswell R (2009).

[b1006] Pikuta EV, Marsic D, Bej A, Tang J, Krader P, Hoover RB (2005). *Carnobacterium pleistocenium* sp. nov., a novel psychrotolerant, facultative anaerobe isolated from permafrost of the Fox Tunnel in Alaska. Int J Syst Evol Microbiol.

[b1007] Reed DW, Fujita Y, Delwiche ME, Blackwelder DB, Sheridan PP, Uchida T, Colwell FS (2002). Microbial communities from methane hydrate-bearing deep marine sediments in a forearc basin. Appl Environ Microbiol.

[b5] Schippers A, Neretin LN, Kallmeyer J, Ferdelman TG, Cragg BA, Parkes RJ, Jorgensen BB (2005). Prokaryotic cells of the deep sub-seafloor biosphere identified as living bacteria. Nature.

[b15] Schultheiss PJ, Francis TJG, Holland M, Roberts JA, Amann H, Thjunjoto, Rothwell RG (2006). Pressure coring, logging and sub-sampling with the HYACINTH system. New Techniques in Sediment Core Analysis.

[b20] Smith DC, Spivack AJ, Fisk MR, Haveman SA, Staudigel H (2000). Tracer-based estimates of drilling-induced microbial contamination of deep sea crust. Geomicrobiol J.

[b48] Süß J, Engelen B, Cypionka H, Sass H (2004). Quantitative analysis of bacterial communities from Mediterranean sapropels based on cultivation-dependent methods. FEMS Microbiol Ecol.

[b51] Tamura K, Dudley J, Nei M, Kumar S (2007). Mega4: molecular evolutionary genetics analysis (MEGA) software, version 4.0. Mol Biol Evol.

[b39] Toffin L, Webster G, Weightman AJ, Fry JC, Prieur D (2004). Molecular monitoring of culturable bacteria from deep-sea sediment of the Nankai Trough, Leg 190 Ocean Drilling Program. FEMS Microbiol Ecol.

[b22] Toffin L, Zink K, Kato C, Pignet P, Bidault A, Bienvenu N (2005). *Marinilactibacillus piezotolerans* sp. nov., a novel marine lactic acid bacterium isolated from deep sub-seafloor sediment of the Nankai Trough. Int J Syst Evol Microbiol.

[b27] Valentine DL (2007). Adaptations to energy stress dictate the ecology and evolution of the *Archaea*. Nat Rev Microbiol.

[b29] Webster G, Newberry CJ, Fry JC, Weightman AJ (2003). Assessment of bacterial community structure in the deep sub- seafloor biosphere by 16S rDNA-based techniques: a cautionary tale. J Microbiol Methods.

[b18] Webster G, Blazejak A, Cragg BA, Schippers A, Sass H, Rinna J (2009). Subsurface microbiology and biogeochemistry of a deep, cold-water carbonate mound from the Porcupine Seabight (IODP Expedition 307). Environ Microbiol.

[b45] Wellsbury P, Goodman K, Barth T, Cragg BA, Barnes SP, Parkes RJ (1997). Deep marine biosphere fuelled by increasing organic matter availability during burial and heating. Nature.

[b2] Whitman WB, Coleman DC, Wiebe WJ (1998). Prokaryotes: the unseen majority. Proc Natl Acad Sci USA.

[b1008] Widdel F, Bak F, Balow A, Trüper HG, Dworkin M, Harder W, Schleifer KH (1992). Gram-negative mesophilic sulfate-reducing bacteria. The Prokaryotes. A handbook on the Biology of Bacteria: Ecophysiology, Isolation, Identification, Applications.

[b11] Yanagibayashi M, Nogi Y, Li L, Kato C (1999). Changes in the microbial community in Japan Trench sediment from a depth of 6292 m during cultivation without decompression. FEMS Microbiol Lett.

[b1009] Zhao JS, Manno D, Hawari J (2007). Abundance and diversity of octahydro-1,3,5,7-tetranitro-1,3,5,7-tetrazocine (HMX)-metabolizing bacteria in UXO-contaminated marine sediments. FEMS Microbiol Ecol.

[b6] Zink KG, Wilkes H, Disko U, Elvert M, Horsfield B (2003). Intact phospholipids – microbial ‘life markers’ in marine deep subsurface sediments. Org Geochem.

